# Astrocyte-secreted factors modulate synaptic protein synthesis as revealed by puromycin labeling of isolated synaptosomes

**DOI:** 10.3389/fnmol.2025.1427036

**Published:** 2025-02-20

**Authors:** Aida de la Cruz-Gambra, Jimena Baleriola

**Affiliations:** ^1^Laboratory of Local Translation in Neurons and Glia, Achucarro Basque Center for Neuroscience, Leioa, Spain; ^2^Neuroscience Department, University of the Basque Country (UPV/EHU), Leioa, Spain; ^3^IKERBASQUE, Basque Foundation for Science, Bilbao, Spain

**Keywords:** local translation, proteins, synaptosomes, astrocyte-secreted factors, puromycilation assays and astrocyte-neuron communication

## Abstract

The synaptic proteome can be shaped by proteins transported from the neuronal soma and/or by mRNAs that are delivered to synapses where proteins are locally synthesized. This last mechanism is known as local translation. Local translation has been extensively studied in neurons in physiological conditions and, more recently, in neurological disorders, in which local transcriptomes and translatomes become dysregulated. It is widely believed that in neurons, the main source of localized transcripts is the neuronal soma and that localized translation is primarily regulated by the neuron itself. However, we wondered whether glial cells, especially astrocytes, could contribute to the modulation of synaptic local protein synthesis. To address this question, we compared levels of proteins produced in synaptic compartments in neuronal and neuron–astrocyte co-cultures using modified Boyden chambers or astrocyte-conditioned medium. We developed a methodology to measure local protein synthesis by puromycin labeling of isolated synaptosomes devoid of somatic input. Our results show that synaptic local translation is enhanced or retained when neurons are cultured in the presence of astrocytes and in response to astrocyte-conditioned medium. Puromycin labeling coupled with proximity ligation identified Rpl26 as one of the proteins whose local synthesis is regulated by astrocyte-secreted factors. Our results thus unravel the contribution of glia to synaptic protein synthesis and point to a previously unexplored extra layer of complexity in the regulation of local translation in neurons.

## 1 Introduction

In the central nervous system (CNS), neuronal connectivity in the brain is accomplished by synaptic connections among neurons (Lynn et al., 2024), which are simultaneously in constant interaction with non-neuronal cells known as glia. Astrocytes are the most abundant glial cells of the CNS, which support neurons with energy through the lactate shuttle, and they regulate processes such as blood flow, axon myelination, long-term memory, and neurotransmitters clearance (Farizatto and Baldwin, 2023; Sun et al., 2024). Astrocytes also contribute to the maintenance and formation of synaptic connections, and they regulate synaptic plasticity through the so-called tripartite synapse (Murai and Pasquale, 2011; Perea et al., 2009). Thus, the constant communication between neurons and astrocytes leads to a proper synaptic function. Neuron–astrocyte communication can occur via direct contact or through secreted factors (Farizatto and Baldwin, 2023; Garrett and Weiner, 2009; Murai et al., 2003; Pyka et al., 2011). The first evidence of astrocytes enhancing synapse formation through secreted factors was observed in primary cultures of purified retinal ganglion cells, where neurons treated with astrocyte-conditioned medium presented more synapses (Ullian et al., 2001). Interestingly, synapse formation, function, and maintenance are partially regulated by local protein synthesis (Leal et al., 2014; Martin, 2004; Yoon et al., 2012; Zhang and Poo, 2002). However, whether astrocytes contribute to local translation in synaptic compartments through secreted molecules is largely unexplored, and it is the focus of this report.

Local translation enables the shaping of local proteomes in neurons, which were originally thought to be maintained by proteins synthesized in the soma and then transported to subneuronal compartments. Local translation requires the delivery of mRNAs rather than proteins to distal subcellular domains (e.g., dendrites, axons, and synapses), where they are locally translated into proteins (Bernard et al., 2022; Hafner et al., 2019; Leal et al., 2014; Liu-Yesucevitz et al., 2011; Wong et al., 2024). This mechanism enables neurons to respond to their environment in an acute manner as proteins are newly produced only where and when they are needed.

Local translation in neurons has been deeply studied in the nervous system under physiological conditions and, more recently in neurodegenerative diseases, in which this mechanism becomes dysregulated (Baleriola et al., 2014; Gamarra et al., 2021). However, there is one question that remains greatly unanswered in this field: Is local protein synthesis in neurons fully regulated by the neuron itself or could non-neuronal cells contribute to this phenomenon to regulate neuronal functions? In this study, we demonstrate that neuron–astrocyte crosstalk through secreted factors regulates local protein synthesis in synapses, which could contribute to synaptic function. Additionally, in this article, we provide a new method to measure local translation by performing puromycin labeling and puromycin-based proximity ligation assays (Puro-PLA) in isolated synaptosomes by immunocytochemical approaches.

## 2 Materials and methods

### 2.1 Animals

All animal protocols followed the European directive 2010/63/EU and were approved by the University of the Basque Country (UPV/EHU) Ethics Committee. Sprague–Dawley rats were bred in local facilities, and embryonic brains (E18) were obtained from CO_2_ euthanized pregnant rats for neuronal and fibroblast cultures, whereas postnatal rats P0-P2 were used for primary astrocytic culture.

### 2.2 Primary neuronal culture

Cultured hippocampal neurons were prepared from embryonic day 18 Sprague–Dawley rat embryos (E18). In brief, hippocampi of rat embryos were dissected in ice-cold 1X Hank’s balanced salt solution (HBSS, Gibco, Thermo Fisher Scientific, Waltham MA, United States). Then, an enzymatic dissociation was performed in 1X TrypLE Express (Gibco) for 10 min at 37°C in a 5% CO_2_ humidified incubator followed by a mechanical homogenization. Cells were centrifuged for 5 min at 200 g, and the resulting pellet was resuspended in plating medium containing filtered Neurobasal medium (Gibco) supplemented with 10% fetal bovine serum (Sigma-Aldrich Aldrich, Merck, Darmstadt, Germany) and 10 U/μL penicillin (Gibco), 10 μg/μL streptomycin (Gibco), and 29.2 μg/μL L-glutamine (Gibco). Hippocampal neurons were plated on poly-D-lysine-coated (Sigma-Aldrich, #P1149) 12-well plates at a density of 100,000 cells/cm^2^ for synaptosomal isolation or 20,000 cells/cm^2^ for puromycilation assay in neurites. Neurons were maintained at 37°C in a 5% CO_2_ humidified incubator. To avoid glial growth, at 1 day *in vitro* (DIV), plating medium was replaced with growth medium containing filtered Neurobasal medium supplemented with 1X B27 (Gibco) and 10 U/μL penicillin, 10 μg/μL streptomycin, and 29.2 μg/μL L-glutamine containing 20 μM of 5-fluorodeoxyuridine (Fdu, Sigma-Aldrich) and 20 μM uridine (Sigma-Aldrich). At 3 DIV, half of the medium was replaced with fresh growth medium supplemented with 20 μM Fdu and 20 μM uridine. At 7 DIV, half of the medium was replaced with growth medium, and neurons were maintained for >21 DIV to ensure mature synapses (Hafner et al., 2019).

### 2.3 Primary astrocytic culture

Primary astrocytes were cultured from mixed glial culture. In brief, brain hemispheres of two postnatal Sprague–Dawley rats (P0-P2) were dissected in 1X HBSS (Gibco). The four hemispheres were placed in a tube containing 4 mL of 1X HBSS (Gibco) and enzymatically dissociated with 0.25% trypsin (Sigma-Aldrich) and 0.004% DNAse (Sigma-Aldrich) during 15 min at 37°C in a 5% CO_2_ humidified incubator. Afterward, the enzymatic dissociation was stopped by adding the same amount of glial plating medium containing IMDM (Gibco), 10% fetal bovine serum Hyclone (Cytiva, Thermo Fisher Scientific), and 10% of a mixture of antibiotics and antimycotics (Gibco). Cells were centrifuged for 6 min at 300 g at room temperature. The pellet was resuspended in 1 mL of glia plating medium and mechanically dissociated using syringes of 21G and 23G needles, respectively. Cells were centrifuged again for 6 min at 300 g at room temperature, and cells were resuspended in 1 mL glia plating medium and seeded onto 75 cm^2^ flasks (BioLite, Thermo Fisher Scientific) and incubated at 37°C in a 5% CO_2_ humidified incubator. Medium was changed to glia medium containing glucose Dulbecco’s Modified Eagle’s Medium (DMEM, Gibco) supplemented with 10% fetal bovine serum (Sigma-Aldrich), 10 U/μL penicillin, 10 μg/μL streptomycin, and 29.2 μg/μL L-glutamine after 1 DIV and every 3 days.

Astrocytes were isolated by agitating the 11 DIV mixed glial culture flasks at 180 rpm for 4 h at 37°C. The medium containing microglial cells was discarded, and the astrocytes attached to the surface of the flask were enzymatically dissociated by adding 7 mL of 1X TrypLE Express (Gibco) for 15 min at 37°C in a 5% CO_2_ humidified incubator. Trypsin reaction was stopped by adding 7 mL of glia medium. Cells were centrifuged for 5 min at 300 g at room temperature. The pellet containing astrocytes was washed ones with 1 mL of growth medium and resuspended in 1 mL of growth medium. For the co-cultures, astrocytes were seeded in a ratio of 1:10 (1 astrocyte: 10 neurons) at the bottom of 1 μm pore Modified Boyden Chambers (Corning, Sigma-Aldrich) previously coated with Poly-D-lysine-coated and co-cultured with 21 DIV hippocampal neurons for 3 days.

### 2.4 Primary fibroblast culture

Primary fibroblast culture was performed from E18 Sprague–Dawley rat embryos ears. Ears of 10 embryos were dissected and then cut into smaller pieces to facilitate their dissociation. The ears were placed in a tube containing 4 mL of 1X HBSS and enzymatically dissociated with 0.25% trypsin and 0.004% DNAse during 30 min at 37°C in a 5% CO_2_ humidified incubator. After 30 min, 4 mL of filtered IMDM supplemented with 10% fetal bovine serum Hyclone and 10% of a mixture of antibiotics and antimycotics was added to stop the enzymatic dissociation. Cells were centrifuged for 6 min at 580 g at room temperature (Khan and Gasser, 2016; Pyka et al., 2011). The pellet was resuspended in 1 mL of the previous supplemented IMDM medium and mechanically dissociated using syringes of 21G and 23G needles, respectively. Fibroblasts were centrifuged again for 6 min at 580 g at room temperature, and cells were resuspended in 1 mL supplemented IMDM medium and seeded onto 75 cm^2^ flasks and incubated at 37°C in a 5% CO_2_ humidified incubator. Medium was changed to DMEM with glucose supplemented with 10% fetal bovine serum, 10 U/μL penicillin, 10 μg/μL streptomycin, and 29.2 μg/μL L-glutamine after 1 DIV and every 3 days.

At 11 DIV, fibroblasts were trypsinized with 7 mL of 1X TrypLE Express for 15 min at 37°C. Subsequently, 7 mL of supplemented DMEM with glucose medium was added to stop trypsin reaction. Fibroblasts were centrifuged for 5 min at 300 g at room temperature, and the pellet containing fibroblasts was washed once with 1 mL of growth medium and resuspended in 1 mL of growth medium. For the co-cultures, fibroblasts were plated in a ratio of 1:10 (1 fibroblast: 10 neurons) at the bottom of 1 μm pore modified Boyden chambers previously coated with Poly-D-lysine-coated and co-cultured with 21 DIV hippocampal neuron for 3 days.

### 2.5 Astrocyte- or fibroblast-conditioned meidum treatment

Astrocytes or fibroblasts were seeded 3 days prior to the treatment day, in 12-well plates as previously described. On the day of the treatment, the conditioned medium (CM) of astrocytes or fibroblasts was collected and immediately placed into 21 DIV hippocampal neurons. Neurons were kept with astrocytic or fibroblast CM for 3 days at 37°C in a 5% CO_2_ humidified incubator. At 24 DIV, a pool of 3 wells of hippocampal neurons were used for each condition to isolate synaptosomes.

### 2.6 *In vitro* synaptosome isolation

Synaptosome isolation was performed using Syn-PER buffer (Thermo Scientific). In brief, neurons were washed twice with cold 1X PBS and of 200 μL/well of Syn-PER reagent supplemented with 1X EDTA-free protease and phosphatase inhibitor (#A32961, Thermo Fisher Scientific) and 0.04 U/μL ribonuclease inhibitor (Fisher BioReagents, Thermo Fisher) was added to each culture well. Neurons were gently detached from the culture substrate with a cell lifter and transferred to a tube at 4°C (a pool from 3 to 5 wells were used). Cell debris was removed by centrifugation at 1,200 g for 10 min at 4°C. The pellet containing nuclear components was discarded, and the supernatant was collected and centrifuged at 20,000 g for 30 min at 4°C. Finally, the supernatant (cytosolic fraction) was reserved for immunoblotting, and the pellet (crude synaptosomal fraction) was resuspended in 40 μL of Syn-PER buffer or PBS for immunoblotting or immunofluorescence studies, respectively.

### 2.7 Cryo-electron microscopy

For the vitrification of the sample, freshly glow-discharged 200-mesh grids (R 3.5/1; QUANTIFOIL) were placed inside the chamber of an EM GP2 Automatic Plunge Freezing (Leica, Wetzlar, Germany), which was maintained at 8°C and relative humidity close to saturation (90% rH). Then, 4 μL of the sample were dropped onto the grid for 30 s. After incubation, most of the liquid on the grid was removed by blotting with absorbent standard filter paper (Ø55mm, Grade 595, Hahnemühle). After the blotting step, the grid was abruptly plunged into a liquid ethane bath, automatically set to −184°C. Once the specimen was frozen, the vitrified grid was removed from the plunger and stored under liquid nitrogen inside a cryo-grid storage box.

Cryo-TEM analysis of the samples was performed on a JEM-2200FS/CR (JEOL Europe) transmission electron microscope. This microscope is equipped with a field emission gun (FEG) operated at 200 kV and an in-column *Ω* energy filter. During imaging, no-tilted zero-loss two-dimensional (2D) images were recorded under low-dose conditions, utilizing the ‘Minimum Dose System (MDS)’ of Jeol software, with a total dose on the order of 30–40 electrons/Å^2^ per exposure, at defocus values ranging from 1.5 to 4.0 μm. The in-column Omega energy filter of the microscope helped us to record images with improved signal-to-noise ratio (SNR) by zero-loss filtering, using an energy selecting slit width of 30 eV centered at the zero-loss peak of the energy spectra. Digital images were recorded in linear mode on a 3,840 × 3,712 (5 μm pixels) Gatan K2 Summit direct detection camera (Gatan Inc.) using DigitalMicrograph^™^ (Gatan Inc.) software, at nominal magnifications of 1,500X and 8,000X with a pixel size of 2.7 nm and 0.49 nm, respectively.

### 2.8 Immunoblotting

Protein quantification of whole lysates, synaptosomal fraction, and cytosolic fraction was carried out with the Pierce BCA Protein Assay Kit (Thermo Fisher), following manufacturer’s instructions. Proteins (3–5 μg) were fractioned by SDS-PAGE electrophoresis under reducing conditions (5% β-mercaptoethanol, Gibco) at a 135 V voltage for 90 min in 1.0 mm 4–12% Tris-glycine gels (Invitrogen, Thermo Fisher Scientific). Samples were then transferred at 30 V for 1 h in a 0.2 μm PVDF blotting membrane (Amersham, Sigma-Aldrich), previously activated with methanol (Fisher BioReagents, Thermo Fisher) for 15 min at room temperature. Proteins were visualized with ponceau (Thermo Fisher), washed in TBS-0.1% Tween20 (TBS-T), and blocked in 5% BSA (Sigma-Aldrich) in TBS-T for 1 h at room temperature (RT). The following primary antibodies, namely, mouse anti-PSD95 (1:1000, 95 kDa, Merck #MAB1596), rabbit anti-NR2A (1:1000, 180 kDa, Merck #AB1555P), rabbit anti-Homer1 (1:500, 45 kDa, Synaptic Systems #160003), mouse anti-synaptophysin1 (1:2000, 38 kDa, BioLegend #837102), and rabbit anti-actin (1:10000, 42 kDa, Sigma-Aldrich #SAB4301137), were incubated in 3% BSA in TBS-T overnight in agitation at 4°C. Membranes were washed three times in TBS-T and incubated with the appropriate secondary HRP-conjugated antibodies for 1 h at RT. Membranes were washed three times in TBS-T, and the signal was detected by West Pico Plus chemiluminescent substrate (Protein biology, Thermo Fisher) using the ChemiDoc imaging system (Bio-Rad, Hercules, CA, United States). Total amount of protein was quantified with amido black staining solution (Sigma-Aldrich).

### 2.9 Pharmacological treatments

For puromycilation assays, cells were treated with 2 μM puromycin dihydrochloride from *Streptomyces alboniger* (Sigma-Aldrich) for 2, 10, or 30 min at 37°C prior to the end of the treatment. Neurites were exposed to 2 min of puromycin, whereas isolated synaptosomes were exposed to 10 min of puromycin, unless otherwise stated. DMSO was used as a vehicle. To block protein synthesis, cells were treated with 40 μM anisomycin dissolved in DMSO (Sigma-Aldrich) for 30 min at 37°C.

Soluble oligomeric Aβ was prepared as previously described (Dahlgren et al., 2002; Gamarra et al., 2020). In brief, synthetic Aβ_1–42_ peptides (Bachem, Bubendorf, Switzerland) were resuspended in dry dimethylsulfoxide (DMSO; 5 mM, Sigma-Aldrich) and Hams F-12 pH 7.4 (PromoCell Labclinics, Barcelona, Spain) to 100 μM final concentration. Peptides were incubated overnight at 4°C. Oligomerized Aβ was added to neurons culture or neuron–astrocyte co-cultures at 23 DIV at a 3 μM concentration and incubated for 24 h at 37°C in a 5% CO_2_ humidified incubator. DMSO was used as vehicle control.

### 2.10 Puromycilation assay

Puromycin is an aminoacyl-tRNA analog that incorporates into nascent polypeptide chains during elongation in a ribosome-catalyzed reaction (Schmidt et al., 2009), and specific anti-puromycin antibodies can be used to detect *de novo* protein synthesis. A total of 24 DIV neurons or neuron–astrocyte co-cultures were exposed to 2 μM puromycin diluted in culture medium for 2 min. To remove puromycin excess, cells were washed once with 1X PBS supplemented with 3 μg/mL of digitonin (Sigma-Aldrich) followed by another wash with 1X PBS. Finally, neurons were fixed with 4% paraformaldehyde (PFA) (Sigma-Aldrich) with 4% sucrose in PBS for 20 min at RT.

**Figure 1:**
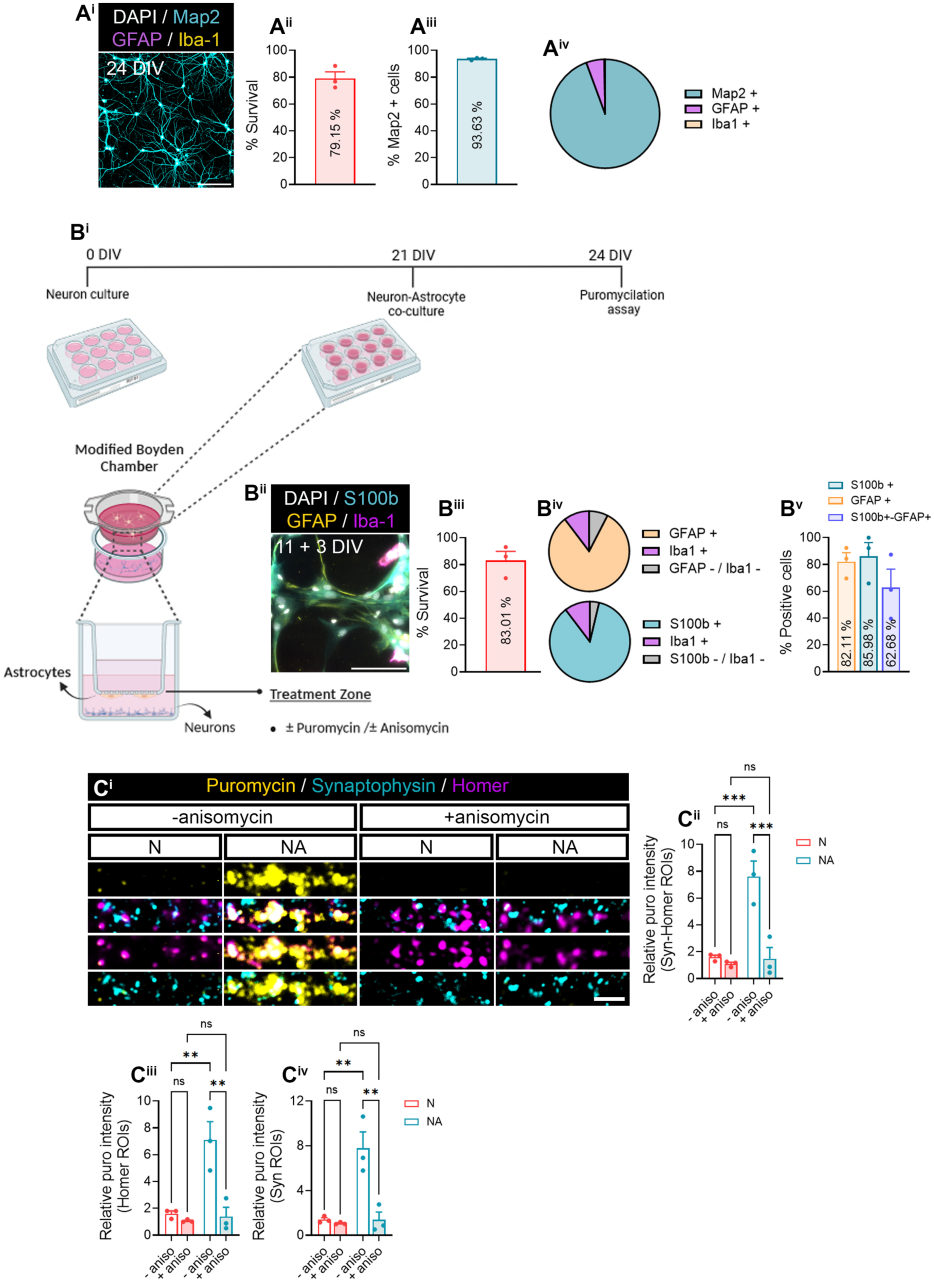
Neuron–astrocyte-secreted factors induce local translation in synaptic compartments. **(A**^**i**^**)** Primary hippocampal neuron cultures were characterized by staining with MAP2 dendritic marker (cyan), GFAP astrocytic marker (magenta), Iba1 microglial marker (yellow), and DAPI (gray). Scale bar 50 μm. Survival of cells is shown in **(A**^**ii**^**)**. The bar graph represents the mean ± SEM of three independent experiments. The percentage of MAP2+ neurons is quantified in **(A**^**iii**^**)**. The bar graph represents the mean ± SEM of three independent experiments. The pie chart **(A**^**iv**^**)** depicts the percentage of neurons (MAP2+) as well as other cell types such as astrocytes (GFAP+) or microglia (Iba1+) observed in neuronal cultures. **(B)** Neurons were cultured in modified Boyden chambers in the presence or absence of astrocytes. **(B**^**i**^**)** Depiction of the culture approach used to culture neurons or to perform neuron–astrocyte co-cultures in modified Boyden chambers. Image created with BioRender. **(B**^**ii**^**)** Astroglial cultures were characterized by staining with S100b astrocytic markers S100b (cyan) and GFAP (yellow), Iba1 microglial marker (magenta), and DAPI (gray). Scale bar 50 μm. Survival of cells is shown in **(B**^**iii**^**)**. The bar graph represents the mean ± SEM of three independent experiments. The pie charts **(B**^**iv**^**)** depict the percentage of astrocytes (S100b or GFAP-positive) as well as microglia (Iba1+) and other undetermined cells (negative for S100b−, GFAP, and Iba−1). The percentage of S100b and/or GFAP-positive cells is quantified in **(B**^**v**^**)**. The bar graph represents the mean ± SEM of three independent experiments. **(C)** Local translation in synaptic compartments is enhanced by the presence of astrocytes in culture. Twenty four DIV neuron cultures (N) or neuron–astrocyte co-cultures (NA) in modified Boyden chambers in were exposed 2-min with 2 μM puromycin. Translation was blocked with 40 μM anisomycin. Representative micrographs are shown in **(C**^**i**^**)**. Bar graphs show changes in puromycin labeling in distinct synaptic compartments in the presence of astrocytes **(C**^**ii**^**–C**^**iv**^**)**. Data were analyzed in three independent cultures and analyzed by two-way ANOVA followed by Holm-Sidak’s *post-hoc* analysis for selected pairs of columns. **p < 0.01; ***p < 0.001; n.s: not significant.

In the case of isolated synaptosomes, these were resuspended in 1 mL 1X PBS and divided into 167 μL aliquots that were transferred to a 24-well plate containing poly-D-lysine-coated 12 mm coverslips. Synaptosomes were exposed to puromycin diluted in PBS for 10 min at 37°C, except for the experiments on Aβ-treated cells in which the exposure was extended to 30 min. For the short exposure to conditioned medium (Figure 1B), puromycilation assays were performed with 2 μM puromycin diluted in astroglia-conditioned medium. In any case, after treatments, the plates were centrifuged at 1,400 g for 30 min at room temperature. Synaptosomes were fixed with 4% PFA and 4% sucrose in PBS for 20 min at room temperature.

To block protein synthesis and whenever stated, both neurons and isolated synaptosomes were pretreated with 40 μM anisomycin for 20–28 (depending on the puromycin pulse duration) min at 37°C prior to the puromycin exposure. Samples treated with neither puromycin nor anisomycin were used as negative controls and subjected to the same procedures as experimental samples.

### 2.11 Immunofluorescence

After fixation, cells or isolated synaptosomes were washed three times with 1X PBS (5 min each wash) and blocked for 30 min in agitation in 3% BSA, 100 mM glycine, and 0.25% Triton X-100 (Thermo Fisher Scientific). Samples were incubated overnight at 4°C with primary antibodies including mouse anti-puromycin (1:500, Merck Millipore #MABE343), chicken anti-synaptophysin 1 (1:500, Synaptic Systems #101006), mouse anti-PSD95 (1:500, Merck #MAB1596), rabbit anti-homer 1 (1:500, Synaptic Systems #160003), guinea pig anti-Homer1 (1:500, Synaptic Systems #160005), rabbit anti-SNAP25 (1:250, Abcam #S9684), and rabbit anti-Rpl26 (1:120, Abcam #ab59567). The following day, after three washes with 1X PBS, cells were incubated with fluorophore-conjugated secondary antibodies Alexa Fluor 594 goat anti-mouse IgG (H + L) (1:200, Invitrogen #A11005), Alexa Fluor 488 goat anti-chicken Ig Y (H + L) (1:200, Abcam #ab150169), Alexa Fluor 647 donkey anti-rabbit IgG (H + L) (1:200, Invitrogen #A31573), and Alexa Fluor 647 goat anti-guinea pig IgG (H + L) (1:200, Invitrogen #A21450) for 1 h at room temperature. Samples were washed three times with 1X PBS and mounted with ProLong Gold Antifade Reagent with DAPI (Invitrogen). Of each secondary antibody, a no-primary-antibody negative control was used.

### 2.12 Proximity ligation assay

Proximity ligation assays (PLA) were performed using Duolink^®^ *In Situ* Red Started Kit Mouse/Rabbit (Sigma-Aldrich #DU092008). In brief, fixed cells or synaptosomes were washed three times with 1X PBS for 5 min and permeabilized with 3% BSA, 100 mM glycine, and 0.25% Triton X-100 (Thermo Fisher Scientific) for 30 min at room temperature in agitation. The blocking was performed by adding a drop of Duolink^®^ Blocking solution (Sigma-Aldrich) into coverslips at 37°C for 1 h. The mix of primary antibodies was diluted in Duolink^®^ antibody diluent (Sigma-Aldrich) and incubated overnight at 4°C. In brief, the presynaptic antibody chicken anti-synaptophysin (1:500, Synaptic Systems #101006) and the postsynaptic antibody guinea pig anti-Homer1 (1:500, Synaptic Systems #160005) were co-incubated with the primary antibodies required for the proximity ligation assay (PLA). On the one hand, newly synthesized Rpl26 proteins (Puro-Rpl26 PLA) were detected by adding mouse anti-puromycin (1:500, Merck Millipore #MABE343) and rabbit anti-Rpl26 (1:120, Abcam #ab59567) antibodies to the mix of synaptic antibodies, while mouse anti-puromycin (1:500, Merck Millipore #MABE343) and rabbit anti-SNAP25 (1:250, Abcam #S9684) were co-incubated with pre- and postsynaptic antibodies to detect the PLA between puromycin and SNAP25 (Puro-SNAP25 PLA).

The following day, coverslips were washed twice with wash buffer A (Sigma-Aldrich) for 5 min, and subsequently, PLA signal was developed following manufacturer’s instructions. In brief, plus PLA (rabbit probe, 1:5, Sigma-Aldrich) and minus PLA (mouse probe, 1:5, Sigma-Aldrich) probes were diluted in Duolink^®^ antibody diluent (Sigma-Aldrich) at 37°C for 60 min. Samples were washed twice with wash buffer A for 5 min. The ligation of both probes was carried out by incubating coverslips in Duolink^®^ ligation buffer 5X (1:5, Sigma-Aldrich) and 1 U/μL ligase (1:40, Sigma-Aldrich) in ddH_2_O at 37°C for 30 min. Before the amplification step, cells were washed twice with wash buffer A and incubated with Duolink^®^ amplification buffer 5X (1:5, Sigma-Aldrich) and 10 U/μL polymerase (1:80, Sigma-Aldrich) in ddH_2_O at 37°C for 100 min. Coverslips were washed twice with wash buffer B (10 min each wash), followed by 10 min wash with 0.01% wash buffer B and a final wash with 1X PBS for 5 min. Samples were incubated at room temperature for 1 h with secondary antibodies for synaptic makers Alexa Fluor 488 goat anti-chicken Ig Y (H + L) (1:200, Abcam #ab150169) and Alexa Fluor 647 goat anti-guinea pig IgG (H + L) (1:200, Invitrogen # A21450). Finally, coverslips were washed three times with 1X PBS and mounted with ProLong Gold Antifade Reagent with DAPI (Invitrogen).

### 2.13 Image acquisition

Images were acquired using an EC Plan-Neofluar 63×/1.4 Oil DIC M27 objective on an Axio Observer Z1 microscope equipped with AxioCam MRm Rev. 3 (Zeiss, Oberkochen, Germany) digital camera. Images of neurites were acquired with 1.6X Optovar., while synaptosomes images were obtained with 1X Optovar. The settings applied for samples were determined in a random field of a control sample and ensuring no intensity saturation. Images from five random fields per coverslip were acquired with ZEN 2 (blue edition) version 2.0.0.0. software (Zeiss). Quantifications performed in neurites (being a neurite a process that extends from the neuronal soma) were performed choosing neurites with an average length of 70 μm.

For survival assessment, images were adjusted for the best fit. Pyknotic nuclei identified by DAPI staining were quantified in 5–10 fields from each sample. Live cells were calculated subtracting the number of apoptotic nuclei to the total amount of cells stained with DAPI and are represented as percentage.

Figure representation has been performed adjusting contrast and background settings. For all images, the setting of the staining of interest was set identically for all conditions, while the pre- and postsynaptic markers used as counterstain were adjusted to obtain an optimal visualization in figures.

### 2.14 Statistical analysis

All the statistical analyses have been carried out with Prism 8 and 10 (GraphPad Software, San Diego, CA, United States). No normality tests were performed prior to statistical analyses. Typically, two-way analyses of variance (ANOVAs) were performed as more than one variable are analyzed. Otherwise, one.way ANOVA or *t*-test analyses were used. Sample size and statistical analyses are specified in the Figure Legends or throughout the Results section.

## 3 Results

### 3.1 Neuron–astrocyte communication through secreted factors induces local translation in synaptic compartments

In this study, we aimed at addressing whether neuron–astrocyte communication regulates local protein synthesis in neurons, specifically in synaptic compartments. Before determining a potential role of astrocytes in synaptic translation, we characterized neuronal monocultures to determine their purity and survival (Figure 1A^i^). Primary hippocampal cultures at 24 days *in vitro* (DIV) exhibited 79.15% of survival (Figure 1A^ii^), with 93.53% of live cells being positive for the neuronal marker MAP2 (Figure 1A^iii^). We identified a 5.38% of GFAP-positive cells and 0.22% of Iba1-positve cells (Figure 1A^iv^). These results indicate a high enrichment of neurons in our primary cultures. On the other hand, 14 DIV primary glial cultures (Figure 1B^ii^) showed 83.01% of survival (Figure 1B^iii^). 85.99% of living cells expressed the astroglial marker S100β, and 82.11% were GFAP-positive (Figure 1B^v^; Supplementary Figure 1A). In addition, based on the percentage of S100β- or GFAP-expressing cells, we estimated an average of 10.32% of cells being Iba1-positive microglia, and 3.70–7.57% could not be identified either as astrocytes or microglia (Figure 1B^iv^, lower and upper pie charts, respectively). Finally, 62.68% of cells were positive for both S100β and GFAP.

After characterizing our primary cultures, we performed neuron–astrocytes co-cultures in modified Boyden chambers, which consist of inserts with a 1 μm-diameter-pore polyethylene terephthalate (PET) membrane, that enable communication between two cell types through secreted factors (Figure 1B^i^). Primary hippocampal neurons were seeded onto coverslips and cultured for 21 DIV, time in which 11 DIV astrocytes were seeded onto the membrane and co-cultured with the neurons for 3 days. Neuron-only cultures were used as controls. To visualize local translation in synaptic compartments, cells were exposed to a 2-min puromycin pulse. Puromycin is an aminoacyl-tRNA analog that incorporates into nascent polypeptide chains during elongation in a ribosome-catalyzed reaction (Schmidt et al., 2009), and specific anti-puromycin antibodies can be used to detect *de novo* protein synthesis. Cells were counterstained with antibodies against synaptophysin-1 (Syn) and Homer-1 (Homer) to visualize pre- and postsynaptic compartments, respectively (Figure 1C^i^). Our results indicated a significant increase in relative puromycin levels in areas covered by the colocalization between pre- and postsynaptic markers (Syn-Homer ROIs; Figure 1C^ii^) in co-cultures compared to neuronal monocultures. These results were likely attributed to the effect of astrocytes on both post- and presynaptic translation, as increased puromycin labeling was observed separately in Homer and Syn ROIs (Figures 1C^iii,iv^). To verify puromycin incorporation was indeed translation-dependent, some cultures were pre-treated with the protein synthesis inhibitor anisomycin 30 min prior to the puromycin pulse. We confirmed that the puromycin labeling observed in synaptic compartments in the presence of astrocytes was in fact a result of increased protein synthesis (Figure 1C). These results suggest that neuron–astrocyte communication promotes local translation in neurons.

**Figure 2:**
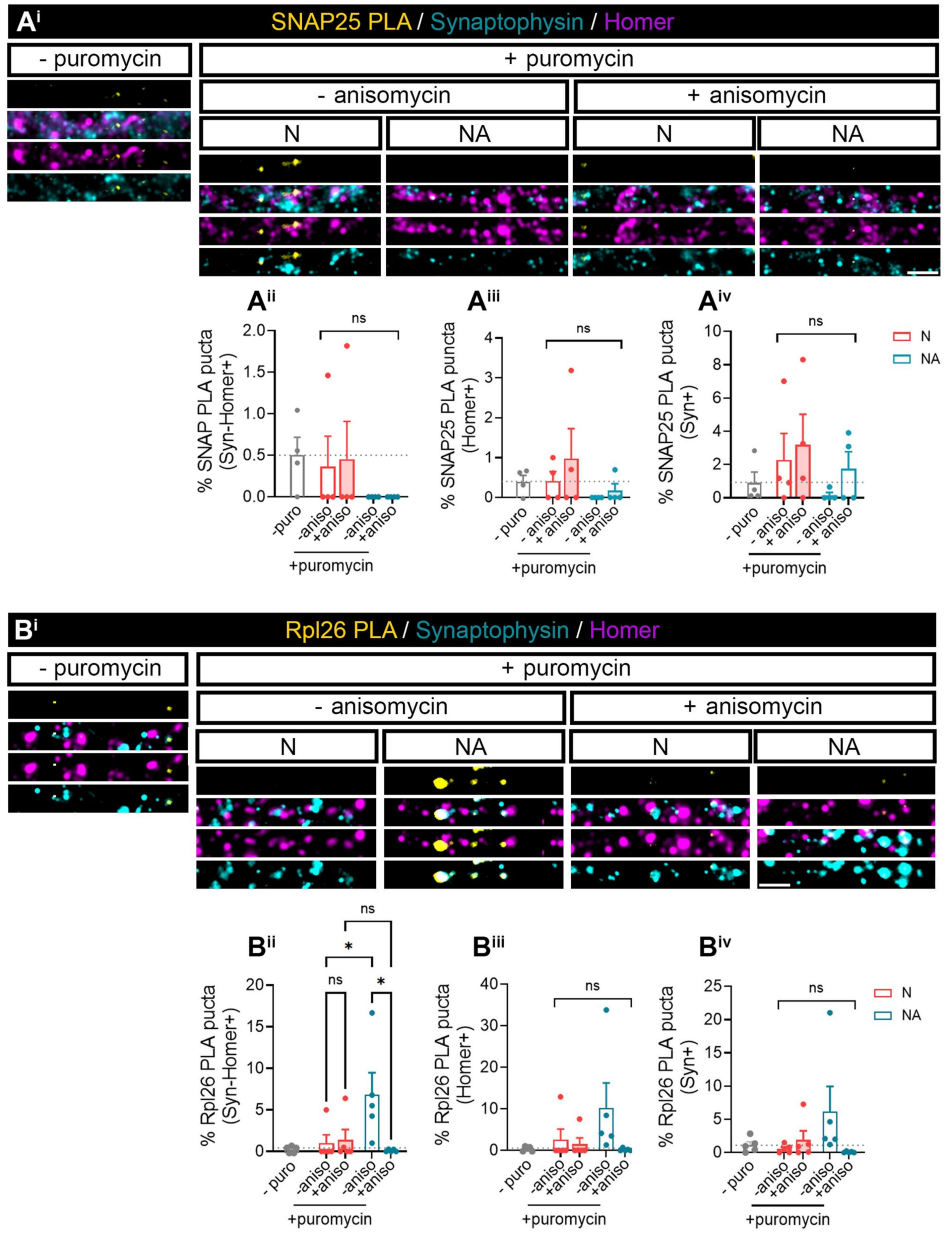
Factors secreted in neuron–astrocyte co-cultures induce *Rpl26* synaptic local translation in basal conditions. Twenty four DIV neuron cultures (N) or neuron–astrocyte co-cultures (NA) in modified Boyden chambers in basal condition were exposed 2-min with 2 μM puromycin. The translation was blocked with 40 μM anisomycin. Cells were treated with vehicle (− puromycin) as negative control. Puromycin proximity ligation assay (Puro-PLA) was performed of **(A**^**i**^**)** SNAP25 protein (SNAP25 PLA) and **(B**^**i**^**)** Rpl26 protein (Rpl26 PLA). Scale bar: 2 μm. Percentage (%) of the triple colocalization analysis obtained from **(A**^**ii**^**)** SNAP25 PLA or **(B**^**ii**^**)** Rpl26 PLA puncta with synaptophysin-Homer+ synapses (Syn-Homer+, obtained as a result of the double colocalization of Syn + and Homer+ puncta), normalized to the total Syn-Homer+ synapse for each individual condition. Percentage of the double colocalization of **(A**^**iii**^**)** SNAP25 PLA or **(B**^**iii**^**)** Rpl26 PLA puncta with Homer postsynapses (Homer+), normalized to the total Homer+ for each individual condition. Percentage of the double colocalization of **(A**^**iv**^**)** SNAP25 PLA or **(B**^**iv**^**)** Rpl26 PLA puncta with Syn + presynapses (Syn+), normalized to the total Syn + for each individual condition. In all graphs, levels of no-puromycin negative controls are shown for descriptive purposes. Two-way ANOVA test was carried out, and whenever the ANOVA was significant (ns when the ANOVA was not significant), Holm-Sidak’s *post-hoc* analysis for selected pairs of columns was performed, *p < 0.05 and ns: not significant. SNAP25 PLA graphs represent mean ± SEM of four independent experiments, whereas Rpl26 PLA graphs represent mean ± SEM of five independent experiments.

We next wanted to investigate whether we were able to find specific proteins whose local synthesis in neurons could be modulated by the presence of astrocytes. Based on the literature, we identified two potential candidates, namely, SNAP25 and Rpl26. On the one hand, there is evidence showing that the synaptosomal protein SNAP25 is locally synthesized in presynaptic terminals during synapse formation *in vitro* (Batista et al., 2017). On the other hand, the mRNA encoding ribosomal protein Rpl26 is known to be locally translated in dendrites (Fusco et al., 2021). Thus, to determine whether these proteins were modulated by the presence of astrocytes, we performed proximity ligation assays (PLA) combining antibodies against puromycin and the protein of interest. We again treated neuronal monocultures or neuron–astrocyte co-cultures with puromycin for 2 min, and we quantified the triple colocalization between the PLA signal, Syn, and Homer (to visualize newly synthesized proteins in synapses) or the double colocalization between the PLA signal and either Homer or Syn (to visualize newly synthesized proteins in post- or presynaptic compartments, respectively). The results were accordingly normalized to the total number of puncta stained with both Syn and Homer, or with either Syn or Homer. No significant SNAP25-PLA signal was detected in synaptic compartments neither in neuronal cultures nor in neuron–astrocytes co-cultures as the identified percentage of positive puncta were similar in cells incubated with puromycin alone or with puromycin and anisomycin (Figures 2A^ii–iv^). Levels of no-puromycin negative controls are shown for descriptive purposes, although one-way ANOVA comparing this column to all other columns confirmed no detection of SNAP25 synthesis: *p* = 0.55 for Syn-Homer+ compartments; *p* = 0.43 for Homer+ compartments; *p* = 0.47 for Syn + compartments). We then wondered whether a neurodegenerative stimulus would uncover an effect of astrocytes on local SNAP25 synthesis. We treated cultures with Aβ oligomers, main drivers of Alzheimer’s disease, which are known to induce local translation in axons (Baleriola et al., 2014; Gamarra et al., 2021). However, we were again unable to detect SNAP25-PLA puncta in neither experimental condition (Supplementary Figure 1B). Conversely, Rpl26 synthesis was readily visible in neurons co-cultured with astrocytes compared to neuronal monocultures (Figure 2B), although only in Homer-Syn-positive synapses (Figures 2B^i–iii^). Thus, we could not attribute this effect to dendritic spines or presynaptic terminals. No effect of astrocytes could be detected in cultures treated with Aβ oligomers (Supplementary Figure 1C). Thus far, our results indicate that communication between astrocytes and neurons through secreted factors enhances the local synthesis of at least Rpl26 in basal conditions, and this effect might be impaired in pathological conditions.

### 3.2 Isolated synaptosomes are functionally competent to incorporate puromycin in a protein synthesis-dependent manner

Puromycin assays described thus far were performed by feeding cultures with puromycin for only 2 min. Given this short exposure, it is unlikely that newly synthesized puromycilated peptides arise in synapses as a result from the transport of somatically produced proteins. However, one of the potential limitations of exposing neurons to such a short pulse is that, depending on the translation rate of localized transcripts, signals arising from the PLA approach might be below detection levels. Hence, we decided to isolate synaptosomes devoid from somatic inputs, and once isolated, we exposed them to puromycin for a longer period to improve the detection of newly synthesized proteins. Our aim was 2-fold: first, to determine whether isolated synaptosomes still retained their translation capacity; second, to confirm that astrocytes modulate synaptic translation in neurons.

**Figure 3:**
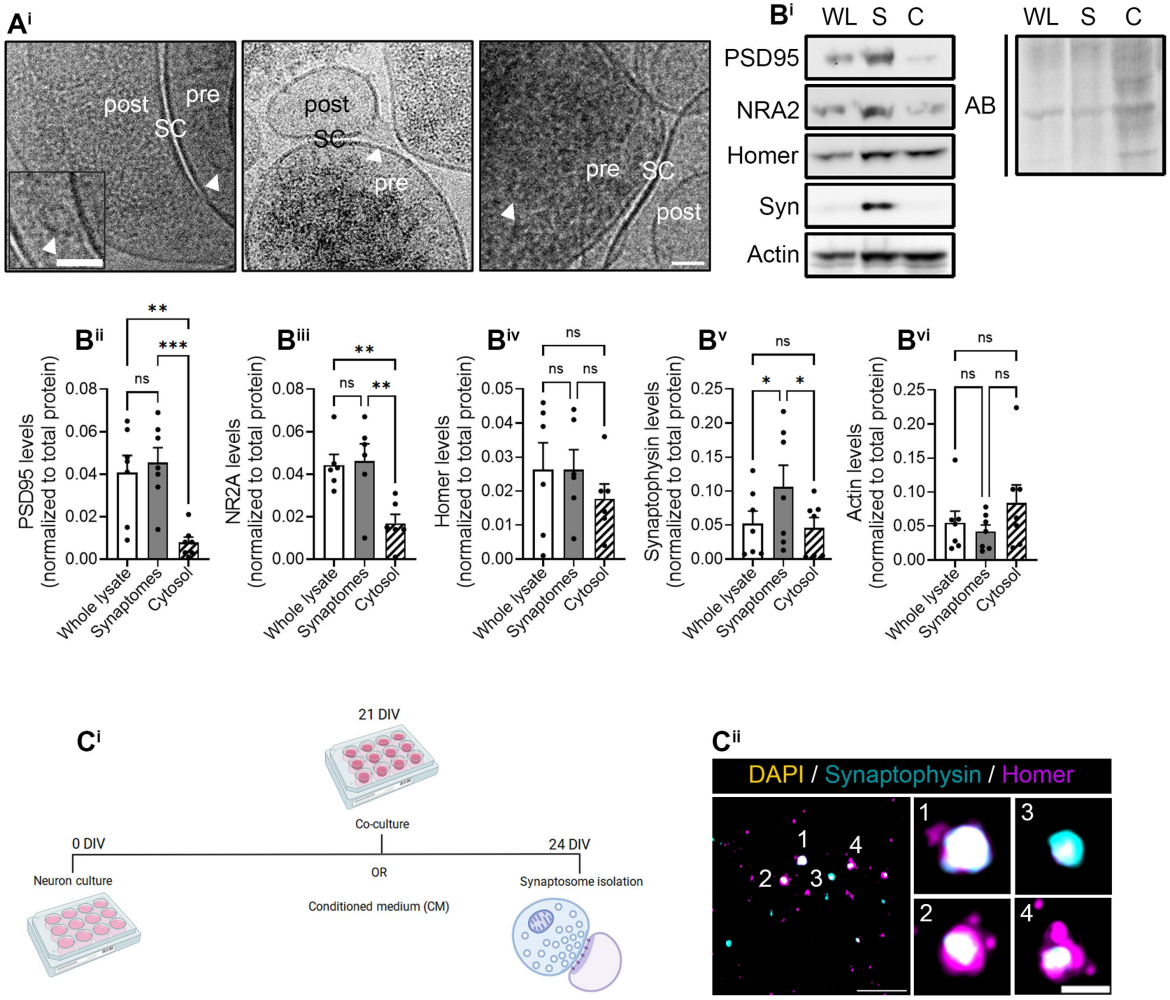
Synaptosome characterization. Synaptosome characterization by **(A**^**i**^**)** Cryo-EM image of isolated synaptosomes, where presynapses (pre) and postsynapses (post) can be distinguished by the synaptic cleft (SC). Some synaptic vesicles (indicated by arrowheads in the images) can be observed within presynaptic compartment. Scale bar 50 nm (25 nm in inset). **(B**^**i**^**)** Representative images of the Western blot (WB). All proteins were normalized to the total amount of protein detected with amido black (AB) staining solution. WL, whole lysate; S, synaptosomal fraction; C, cytosolic fraction. WB quantification of **(B**^**ii**^**)** postsynaptic marker PSD95, **(B**^**iii**^**)** N-methyl-D-aspartate (NMDA) receptor (NRA2), **(B**^**iv**^**)** postsynaptic marker Homer-1 (Homer), **(B**^**v**^**)** presynaptic protein synaptophysin-1 (Syn), and **(B**^**vi**^**)** the cytoskeletal marker actin. All proteins were normalized to the total amount of protein. RM one-way ANOVA followed by Holm-Sidak’s *post-hoc* test for selected pairs of columns, *p < 0.05, **p < 0.01, ***p < 0.001, and ns: not significant. **(C**^**i**^**)** Experimental protocol to perform the puromycilation assay in isolated synaptosomes. The representative figure has been created with BioRender. **(C**^**ii**^**)** Immunocytochemistry of isolated synaptosomes represented by synaptophysin-positive pre-synapses (in cyan) and homer-positive postsynapses (in magenta). Synaptosomes were counterstained with DAPI (in yellow) to confirm the absence of the somatic input. Scale bar 10 μm; 2 μm in insets.

We first characterized synaptosomes isolated from hippocampal neurons with Syn-PER buffer by cryo-electron microscopy (Cryo-EM). Cryo-EM evidenced electrodense presynaptic terminals, some of them containing visible synaptic vesicles, with a nearby postsynaptic density, both separated by the synaptic cleft (Figure 3A^i^). Moreover, we identified synaptic proteins by immunoblotting and observed that postsynaptic markers PSD95 and NR2A, although not enriched in synaptosome preparations compared to the whole lysate, they were decreased in the cytosolic fraction (Figures 3B^i–iii^). Conversely, the presynaptic marker Syn was enriched in crude synaptosome preparations compared to both the whole lysate and the cytosol (Figure 3B^v^). Actin was used as a cytoskeletal marker and remained unchanged in all fractions (Figure 3B^vi^). These results were not unexpected as previous publications have reported the enrichment of presynaptic terminals compared to postsynaptic densities using alternative synaptosome isolation methods (Hafner et al., 2019). Finally, we also characterized our synaptosome preparation by conventional immunocytochemistry. To that end, we attached freshly resuspended synaptosomes to poly-D-lysine-treated coverslips by centrifugation. After fixation, synaptosomes were immunostained with antibodies against Syn and Homer, following the same approach as in neuronal cultures (Figures 1, 2). Despite Homer not being enriched in synaptosomes based on our results from immunoblotting (Figure 3B^iv^), we did observe the colocalization of both markers (Figure 3C^ii^), in line with the results obtained by Cryo-EM.

Next, we addressed if isolated synaptosomes were functionally competent to incorporate puromycin into newly synthesized polypeptide chains. Thus, we performed control experiments exposing cells to Aβ oligomers, which induce local translation in axons and presynaptic terminals (Baleriola et al., 2014). Synaptosomes were isolated from vehicle- or Aβ-treated neurons, and, once attached to a coverslip, they were exposed to 2 μM puromycin and/or to the translation inhibitor anisomycin for 30 min. Puromycilation assays revealed a higher colocalization between puromycin and synaptic markers in Aβ-treated cells compared to anisomycin-treated synaptosomes, whereas no differences were observed in synaptosomes isolated from control cells (Supplementary Figure 2A). These results indicate that local translation can be measured in isolated synaptosomes that do not receive somatic input, at least under certain conditions.

### 3.3 Astrocyte-conditioned medium induces local translation in isolated synaptosomes

**Figure 4:**
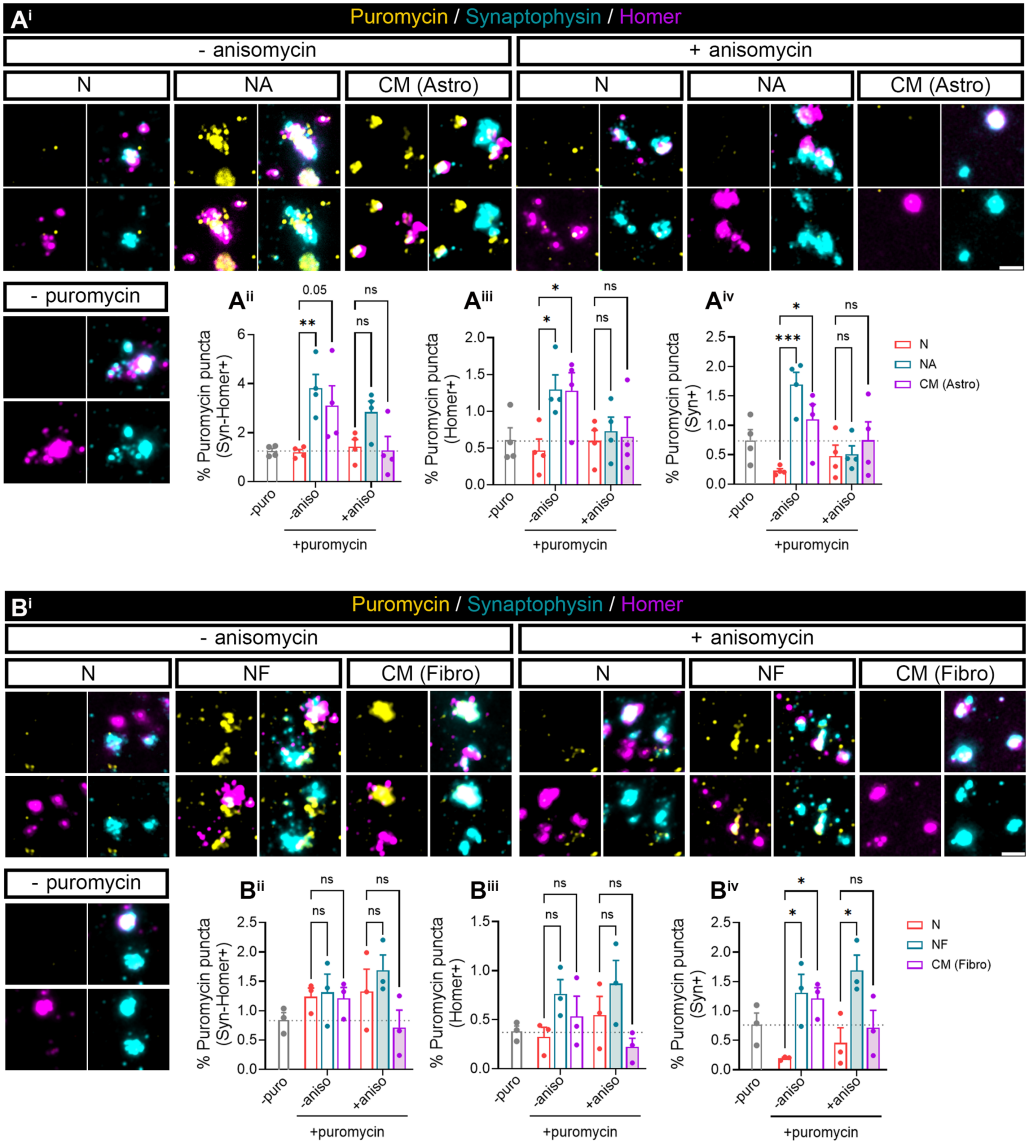
Puromycilation assay detection in isolated hippocampal synaptosomes. Twenty four DIV hippocampal neurons were cultured or co-cultured with astrocytes or astrocytic-conditioned medium **(A)** or co-cultured with fibroblast or fibroblast derived conditioned medium **(B)** for 3 days. **(A**^**i**^**)** Puromycin treatment was carried out in isolated synaptosomes coming from neurons exposed to astrocyte-secreted factors. Scale bar 2 μm. Percentage (%) of the colocalization analysis obtained from puromycin puncta with **(A**^**ii**^**)** synaptophysin-Homer+ synapses (obtained as a result of the double colocalization of Syn + and Homer+ puncta), **(A**^**iii**^**)** Homer postsynapses (Homer+), and **(A**^**iv**^**)** Syn + presynapses (Syn+). Each individual condition was normalized to the total amount of Syn-Homer+ synapses, Homer+ postsynapses, or Syn + presynapses, respectively. Two-way ANOVA followed by Holm-Sidak’s *post-hoc* test for selected pairs of columns, *p < 0.05, **p < 0.01, ***p < 0.001, and ns: not significant (*p* = 0.05). All graphs represent mean ± SEM of four independent biological experiments. **(B**^**i**^**)** Puromycin treatment was performed in isolated synaptosomes coming from neurons in the presence of fibroblast-secreted factors. Scale bar 2 μm. Percentage (%) of the colocalization analysis obtained from puromycin puncta with **(B**^**ii**^**)** synaptophysin-Homer+ synapses (obtained as a result of the double colocalization of Syn + and Homer+ puncta), **(B**^**iii**^**)** Homer postsynapses (Homer+), and **(B**^**iv**^**)** Syn + presynapses (Syn+). Each individual condition was normalized to the total amount of Syn-Homer+ synapses, Homer+ postsynapses, or Syn + presynapses, respectively. Two-way ANOVA statistical test was performed, *p<0.05, and ns: not significant. All graphs represent mean ± SEM of three independent biological experiments.

Our previous results (Figure 1) indicated that neuron–astrocyte communication via secreted factors enhances local protein synthesis in synaptic compartments in neurons. Next, we wanted to determine whether our results could be validated in isolated synaptosomes. To that end, we performed neuronal monocultures on neuron–astrocytes co-cultures in modified Boyden chambers as before. Synaptosomes were then isolated and treated with puromycin for 10 min (Figure 4A^i^). In accordance with our previous observations, the presence of astrocytes increased newly synthetized proteins in Syn-Homer synapses (Figure 4A^ii^), as well as in post- (Figure 4A^iii^) and presynaptic compartments (Figure 4A^iv^). In all cases, this effect was blocked by anisomycin. We also wondered whether factors secreted solely by astroglia would elicit the same effect on synaptic translation such as neuron–astrocyte co-cultures. We therefore exposed neurons to astrocyte-conditioned medium (CM) for 3 days and observed a strong trend toward an increase of puromycin puncta in Syn-Homer synaptosomes (*p* = 0.05; Figure 4A^ii^), which became significant in post- (Figure 4A^iii^) and presynaptic compartments (Figure 4A^iv^). Finally, we wanted to determine whether synaptic translation was selectively enhanced by astrocytes, or whether other cell types would drive a similar response in neurons. Thus, we performed experiments on neurons cultured in Boyden chambers, but we now co-cultured them with fibroblasts or treated them with fibroblast-conditioned medium (Supplementary Figure 2B). Synaptosomes were isolated and exposed to puromycin for 10 min. In this case, neither the presence of fibroblasts in culture nor their conditioned medium had any effect on local translation in Syn-Homer synaptosomes or in postsynaptic compartments (Figures 4B^i–iii^). However, we did observe a significant increase in presynaptic compartments form neurons co-cultured with fibroblasts or exposed to conditioned medium. Interestingly, puromycin incorporation was blocked by anisomycin in the latter but not in synaptic terminals isolated from co-cultured neurons (Figure 4B^iv^). A potential explanation for these results will be discussed later in this report, but we can affirm that fibroblast-conditioned medium enhances local protein synthesis in presynaptic terminals.

In summary, astrocyte-secreted factors regulate synaptic local translation. This effect is not exclusively driven by astrocytes since secreted factors from other cell types, such as fibroblasts, can also modulate newly synthetized proteins at least within the presynaptic compartment. However, both cell types do show differences in their effect on local protein synthesis in synapses and postsynaptic density, which seem to be modulated selectively by astrocytes.

### 3.4 Astrocyte-secreted factors enhance Rpl26 local synthesis in postsynaptic compartments

**Figure 5:**
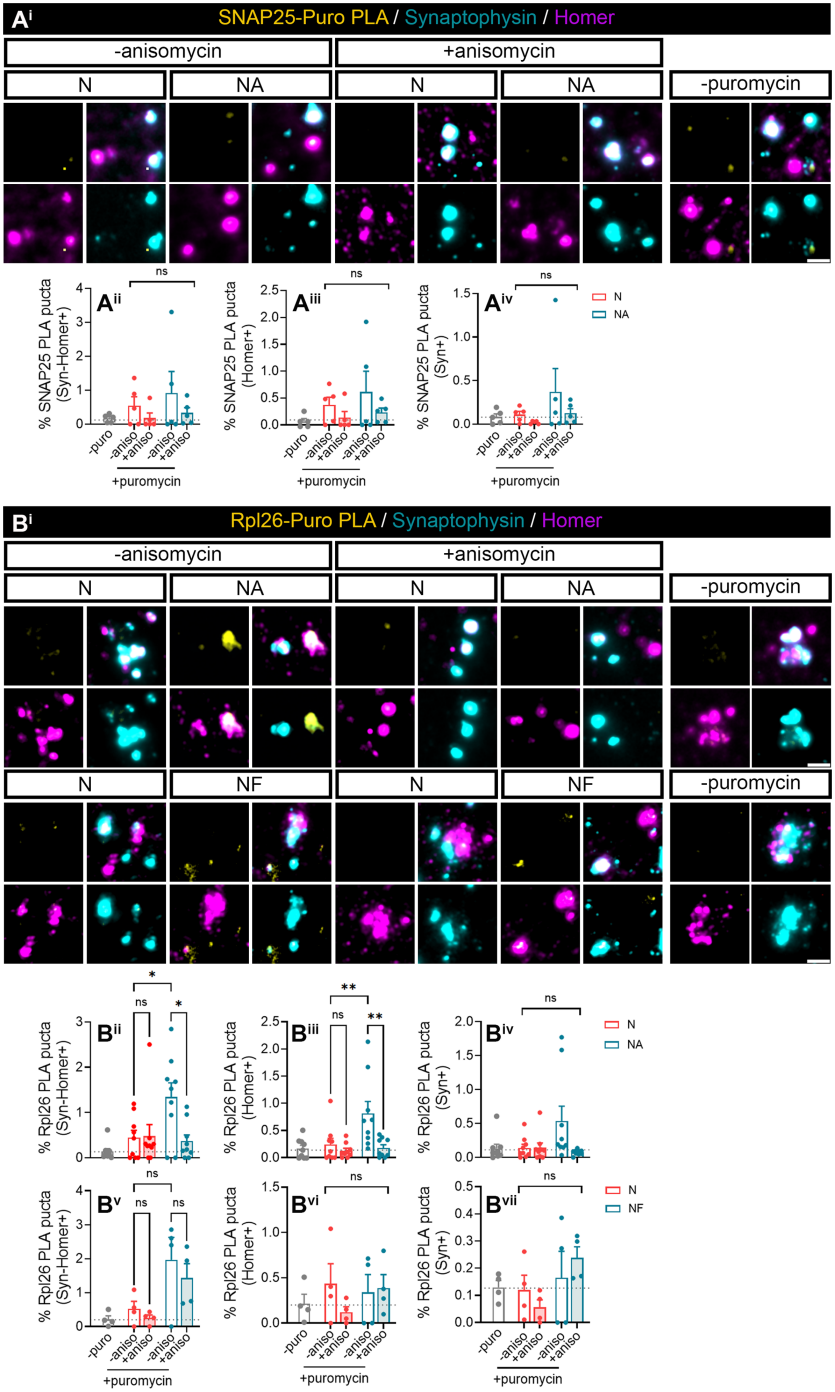
Puromycin direct treatment in isolated synaptosomes confirmed the role of astrocytic-secreted factors to promote *Rpl26* synaptic local translation in basal conditions. Synaptosomes isolated from 24 DIV neuron cultures (N) or neuron–astrocyte co-cultures (NA) in modified Boyden chambers in basal condition were exposed 10-min with 2 μM puromycin. The translation was blocked with 40 μM anisomycin. Cells were treated with vehicle (− puromycin) as negative control. **(A**^**i**^**)** Puro-PLA of SNAP25 (SNAP25 PLA) was performed. Scale bar 2 μm. Percentage (%) of the colocalization analysis obtained from SNAP25 PLA puncta with **(A**^**ii**^**)** synaptophysin-Homer+ synapses (obtained as a result of the double colocalization of Syn + and Homer+ puncta), **(A**^**iii**^**)** Homer postsynapses (Homer+), and **(A**^**iv**^**)** Syn + presynapses (Syn+). Each individual condition was normalized to the total amount of Syn-Homer+ synapses, Homer+ postsynapses, or Syn + presynapses, respectively. In all graphs, levels of no-puromycin negative controls are shown for descriptive purposes. Two-way ANOVA, ns: not significant. All graphs represent mean ± SEM of five independent biological experiments. **(B**^**i**^**)** Puro-PLA of Rpl26 (Rpl26 PLA) was performed in isolated synaptosomes derived from 24 DIV neuron cultures (N) or co-cultures of neuron–astrocyte (NA) or neuron–fibroblast (NF) in modified Boyden chambers in basal condition. Synaptosomes were exposed to a 10-min puromycin pulse and anisomycin for 30 min. Non-puromycin treatment (− puromycin) was used as negative control. Scale bar 2 μm. Percentage (%) of the colocalization analysis obtained from Rpl26 PLA puncta with **(B**^**ii**^**,B**^**v**^**)** synaptophysin-Homer+ synapses (obtained as a result of the double colocalization of Syn + and Homer+ puncta), **(B**^**iii**^**,B**^**vi**^**)** Homer postsynapses (Homer+), and **(B**^**iv**^**,B**^**vii**^**)** Syn + presynapses (Syn+). Each individual condition was normalized to the total amount of Syn-Homer+ synapses, Homer+ postsynapses, or Syn + presynapses, respectively. In all graphs, levels of no-puromycin negative controls are shown for descriptive purposes. Two-way ANOVA test was carried out, and whenever the ANOVA was significant (ns when the ANOVA was not significant), Holm-Sidak’s *post-hoc* test for selected pairs of columns was performed, *p < 0.05, **p < 0.01, and ns: not significant. All graphs represent mean ± SEM of nine independent biological experiments for NA condition, whereas for NF condition four independent biological experiments have been used.

In this study, we aimed at addressing if detection of local SNAP25 and Rpl26 synthesis was improved by exposing isolated synaptosomes with puromycin for 10 min. Like in previous experiments, Puro-SNAP25-PLA puncta were not detected in any synaptic compartment analyzed neither in the present or absence of astrocytes in basal conditions (Figure 5A^i^) nor in response to Aβ treatments (Supplementary Figure 3A). Conversely, Rpl26 synthesis was enhanced by the presence of astrocytes in culture in Syn-Homer synapses (Figures 5B^i,ii^), in line with previous observations. Importantly, the 10-min exposure of isolated synaptosomes with puromycin uncovered the modulation of newly produced Rpl26 in postsynaptic compartments (Figures 5B^i,iii^) although not in presynaptic terminals (Figures 5B^i,iv^). Fibroblasts, on the other hand, did not affect the local synthesis of Rpl26 in Syn-Homer synapses (Figure 5B^v^, Two-way ANOVA, column factor *p*=0.002. No diferences dcetected in *post hoc* test) or in post- and presynaptic compartments (Figures 5B^vi,vii^). Thus, local Rpl26 production is likely selectively boosted by astrocytes in basal conditions. Nevertheless, in response to Aβ treatment Rpl26 local translation was not detected in any synaptic compartment (Supplementary Figure 3B).

**Figure 6:**
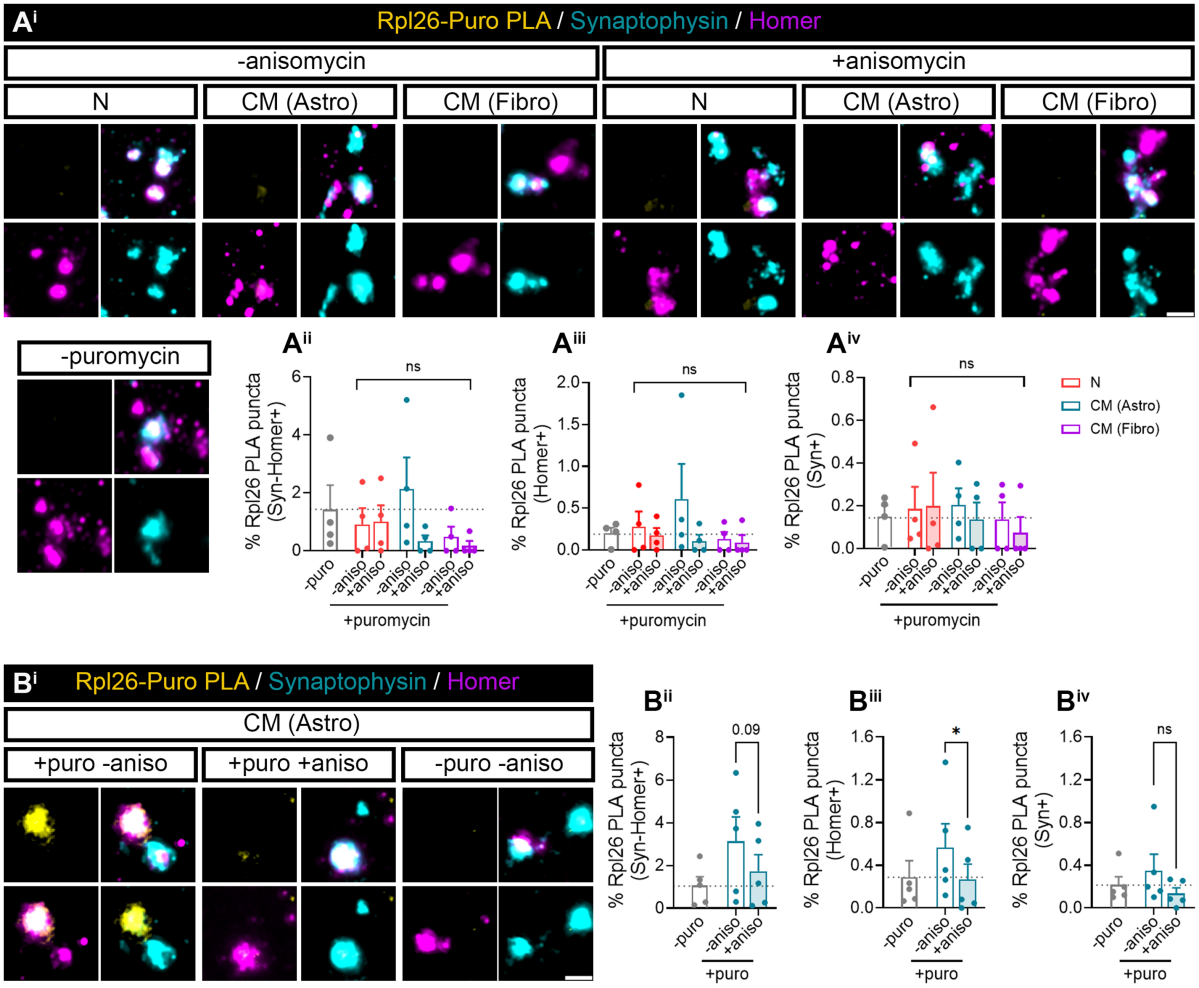
*Rpl26* synaptic local translation is translated in a fast speed manner after astrocyte-conditioned medium treatment in basal conditions. Twenty four DIV hippocampal neurons were treated with neuronal (N), astrocytic (astro), or fibroblast (fibro) conditioned medium (CM). Isolated synaptosomes were treated with 2 μM puromycin for 10 min. The translation was blocked with 40 μM anisomycin for 30 min. Cells were treated with vehicle (− puromycin) as negative control. **(A**^**i**^**)** Puromycin proximity ligation assay (Puro-PLA) was performed of Rpl26 protein (Rpl26 PLA). Scale bar 2 μm. Percentage (%) of the colocalization analysis obtained from Rpl26 PLA puncta with **(A**^**ii**^**)** synaptophysin-Homer+ synapses (obtained as a result of the double colocalization of Syn + and Homer+ puncta), **(A**^**iii**^**)** Homer postsynapses (Homer+), and **(A**^**iv**^**)** Syn + presynapses (Syn+). Each individual condition was normalized to the total amount of Syn-Homer+ synapses, Homer+ postsynapses, or Syn + presynapses, respectively. Two-way ANOVA statistical test was performed, ns: not significant. All graphs represent mean ± SEM of four independent biological experiments. **(B**^**i**^**)** Rpl26 PLA was carried out in isolated synaptosomes directly treated with astrocyte-CM for 30 min. 10 min prior to the end of the treatment, puromycin was added to synaptosomes. Anisomycin was used to inhibit the translation. Scale bar 2 μm. Percentage (%) of the colocalization analysis obtained from Rpl26 PLA puncta with **(B**^**ii**^**)** synaptophysin-Homer+ synapses (obtained as a result of the double colocalization of Syn + and Homer+ puncta), **(B**^**iii**^**)** Homer postsynapses (Homer+), and **(B**^**iv**^**)** Syn + presynapses (Syn+). Each individual condition was normalized to the total amount of Syn-Homer+ synapses, Homer+ postsynapses, or Syn + presynapses, respectively. One-way ANOVA test with Holm-Sidak’s *post-hoc* analysis, *p < 0.05, and ns: not significant (*p* = 0.09). All graphs represent mean ± SEM of five independent biological experiments.

Finally, we wanted to determine whether Rpl26 synthesis was also enhanced by astroglial-conditioned medium. Surprisingly, after 3 days of astrocyte-CM treatment, Puro-Rpl26 PLA puncta were not detected in any synaptic compartment. We obtained similar results with fibroblast CM (Figure 6A). We reasoned that increased synaptic translation observed in synaptosomes in neurons co-cultured with astrocytes could be a result of constant communication between both cell types, in which astroglia continuously secrete molecules to the medium, whereas signals potentially responsible for *de novo* Rpl26 production could be depleted from the conditioned medium over time, hence diminishing their effect. To test this possibility, we acutely exposed synaptosomes to astrocyte-CM while performing puromycin labeling for 10 min. Interestingly, we found that upon direct treatment with astrocyte-CM, Syn-Homer synapses showed a trend toward increasing locally synthesized Rpl26 when compared to synaptosomes co-incubated with anisomycin (Figures 6B^i,ii^, *p* = 0.09). Differences between both conditions were significant when only focusing on double colocalization of Puro-Rpl26 PLA signal with Homer in postsynaptic densities (Figure 6B^iii^), while no changes were detected in presynaptic terminals (Figure 6B^iv^). These results strongly suggest that astrocyte-conditioned medium positively regulates local Rpl26 production at the postsynaptic level in an acute manner.

### 3.5 Puro-PLA labeling of isolated synaptosomes might help identify the global or local origin of synaptic proteins

Finally, we wanted to address if levels of identified proteins (namely, Rpl26 and SNAP25) were changed overall in synaptosomes isolated from neuron–astrocyte co-cultures compared to neuronal monocultures. To our surprise, no changes were detected (Supplementary Figure 4). With these results, it is tempting to speculate that Puro-PLA labeling of isolated synaptosomes might help dissect the relative contribution of locally synthesized proteins versus preexisting protein pools in synaptic compartments.

## 4 Discussion

### 4.1 Potential regulation of local mRNA translation in neurons by cell non-autonomous mechanisms

To our knowledge, this is the first report that directly addresses astroglial regulation of local translation in neurons. We have demonstrated that neuron–astrocyte communication and astrocytic-seceted factors upregulate local protein synthesis in synaptic compartments. Interestingly, fibroblast-conditioned medium also enhanced local protein synthesis in presynaptic terminals. These results are not unexpected, as fibroblast growth factor (FGF) has been historically used to maintain neuronal survival in culture and supports neuritic outgrowth (see Walicke et al., 1986 as example), and local translation is known to promote axon elongation (Leung et al., 2006). One surprising result, however, was that enhanced puromycin labeling in presynapses in neuron–fibroblast co-cultures compared to neuronal monocultures was not inhibited by anisomycin. It has been reported that stalled ribosomes are able to incorporate puromycin, while this reaction is independent of translation elongation inhibitors, such as anisomycin and others (Graber et al., 2013). Thus, one possibility is that, through unknown mechanisms, fibroblast–neuron communication leads to presynaptic ribosome stalling. The functional significance of this phenomenon should be further explored. Overall, our results point toward a cell non-autonomous modulation of the synaptic proteome through local translation in subneuronal compartments. The molecular mechanisms leading to the effect of non-neuronal cells on neurons deserve future investigation. In the case of neuron–astrocyte communication, our results open new exciting venues for the understanding of how astrocytes regulate synaptic function, which is (at least) partially regulated by mRNA localization and local protein synthesis.

### 4.2 Isolated synaptosomes maintain a functional translation machinery

In this study, we implemented a simple methodology to measure local translation in isolated synaptosomes, which lack somatic input, by basic immunocytochemistry. We found that isolated synaptosomes are capable of puromycin incorporation in a protein synthesis-dependent manner. With this approach, we aimed at determining whether isolated synaptosomes still retained their translational capacity. In addition, we wanted to corroborate the influence of astrocytes on local translation in neurons. Our first approaches used 2-min puromycin pulses in neurons (Figures 1, 2) to minimize the diffusion from somatic-derived proteins. However, puromycin assays used to detect local translation have been recently criticized (Enam et al., 2020) even when using short exposures to the drug. Moreover, one of the potential limitations of exposing neurons to such a short pulse is that, depending on the translation rate of localized transcripts, some newly synthesized proteins might not be efficiently detected. Indeed, our own results indicate that Rpl26 localized synthesis was not conclusive in postsynapses when neurons were exposed to puromycin for 2 min in neuron–astrocyte co-cultures compared to monocultures. Conversely, 10-min puromycin treatments of isolated synaptosomes revealed an effect of astroglia on Rpl26 local production in postsynaptic densities. Hence, our approach on isolated synaptosomes uncovered the influence of astrocytes on Rpl26 local postsynaptic synthesis.

### 4.3 Advantages of puromycin labeling of isolated synaptosomes

Neuronal local translation has been addressed in many instances by labeling neurons with non-canonical amino acids or by puromycin tagging of nascent polypeptides (to mention but a few approaches) (Gamarra et al., 2021; Holt et al., 2019), followed by the detection of these molecules in distal neuronal compartments. Short exposure of neurons to such molecules minimizes the potential “contamination” of somatically synthesized proteins transported (or diffused) toward the periphery of neurons. However, recent evidence suggests that even short treatments with puromycin, for instance, might not accurately distinguish local translation from other events (Enam et al., 2020). Synaptosome isolation followed by protein synthesis detection might be a powerful tool to identify local translatomes in synaptic compartments, given that they are disconnected from somatic inputs, which enables labeling of newly synthesized proteins at a *de bona fide* local level. In addition, having the opportunity to treat isolated synaptosomes with puromycin (and similar molecules) at different exposure times might help distinguish fast translating versus slow translating localized transcripts. Indeed, mRNAs encoding ribosomal proteins have been identified as highly translated yet fast decaying mRNAs in axons (Jung et al., 2023). This could explain why long exposure on neurons to astrocyte-conditioned medium enhances overall puromycin incorporation in synaptic compartments, yet Rpl26 local synthesis is only detected upon acute exposure to astroglial-secreted factors.

Although in many instances, mRNA localization in subneuronal compartments has been used a proxy for local translation, local transcriptomes are unlikely a reflection of local proteomes (Jung et al., 2023). In this context, we believe our methodology might help identify which transcripts are locally translated in certain experimental conditions and perform correlation analyses with preexisting localized mRNAs and proteins and even evaluate mRNA local decay and protein half-lives at subcellular levels.

Although this methodology can be perfectly explored in synaptosomes isolated from an entire brain *in vivo*, we believe our *in vitro* approach has the advantage of controlling the cell types that influence neurons (and vice versa) in a controlled environment. The intricate brain connectivity is often difficult to dissect, and our method enables us to test the uni- and bidirectional communication between cell type “pairs.” Obviously, cell-to-cell communication is much more complex in the brain, and this complexity should be taken into consideration.

In addition, our results can be associated with excitatory synapses as we have focus on Homer+ postsynapses, which are enriched in glutamatergic postsynapses. Indeed, astrocyte-secreted factors have been known not only to regulate excitatory synapses but also inhibitory synaptogenesis, where astrocyte-secreted neurocan controls inhibitory synaptogenesis and functions (Irala et al., 2024). Hence, as reported in previous articles, in our synaptosomal preparation, we observed 2.15 times synapses in neuron–astrocyte co-cultures (19,888,027 synaptosomes/mL; from an average of 3,314,617 synaptosomes/cm^2^ obtained in 1/6 mL) compared to the synapses coming from neuron cultures (9,229,510 synaptosomes/mL; from an average of 1,538,241 synaptosomes/cm^2^ in 1/6 mL). Overall, these findings suggest that if astrocyte-secreted factors promote synaptogenesis in excitatory and inhibitory synapses, leading to synaptic proteomic changes, astrocyte-secreted factors could be driven synaptogenesis by local translation in excitatory and inhibitory synapses.

## 5 Conclusion

In this article, we have demonstrated for the first time that astrocyte-secreted factors enhance synaptic mRNA local translation in synaptic compartments. In addition, we have defined a method to visualize local protein synthesis in isolated synaptosomes. We believe this method can be implemented to perform high-throughput analyses of local translatomes in diverse experimental conditions by capturing puromycilated peptides in isolated synaptosomes at different timepoints, as well as to verify locally synthesized proteins by conventional immunocytochemistry. Crude synaptosome lysates can be further separated into presynaptic and postsynaptic fractions, thus distinguishing local presynaptic and postsynaptic translatomes. Both our main finding of neuronal local translation regulation by potential cell-non-autonomous mechanisms and our methodology to measure local protein synthesis in isolated synaptosomes might open new venues in the field of local translation.

## Data Availability

The raw data supporting the conclusions of this article will be made available by the authors upon reasonable request.

## Supplementary material

Supplementary Figure 1 Astrocyte-secreted factors effect on *Rpl26* synaptic local translation is inhibited in Aβ conditions. The expression of S100β and GFAP from cultures used for these experiments is shown **(A**^**i**^**)**. Scale bar, 100 μm. 24 DIV vehicle- or Aβ-treated neuron cultures (N) or Aβ-treated neuron-astrocyte co-cultures (NA) in modified Boyden chambers were exposed 2-minutes with 2 μM puromycin. Anisomycin was used to block translation and no-puromycin treatment (- puromycin) as negative control. Percentage (%) of the colocalization analysis obtained from SNAP25 PLA puncta with **(B**^**i**^**)** synaptophysin-Homer+ synapses (obtained as a result of the double colocalization of Syn+ and Homer+ puncta), **(B**^**ii**^**)** Homer postsynapses (Homer+) and **(B**^**iii**^**)** Syn+ presynapses (Syn+). Each individual condition was normalized to the total amount of Syn-Homer+ synapses, Homer+ postsynapses or Syn+ presynapses, respectively. Two-way ANOVA, ns not significant. All graphs represent mean ± SEM of 4 independent biological experiments. Representative micrographs are shown in **(B**^**iv**^**)**. Scale bar 2 μm. Percentage (%) of the colocalization analysis obtained from Rpl26 PLA puncta with **(C**^**i**^**)** synaptophysin-Homer+ synapses, **(C**^**ii**^**)** Homer+ postsynapses and **(C**^**iii**^) Syn+ presynapses. Each individual condition was normalized to the total amount of Syn-Homer+ synapses, Homer+ postsynapses or Syn+ presynapses, respectively. Representative micrographs are shown in **(C**^**iv**^**)**. Scale bar 2 μm. Two-way ANOVA, ns not significant. All graphs represent mean ± SEM of 5 independent biological experiments. In all graphs levels of no-puromycin negative controls are shown for descriptive purposes.

Supplementary Figure 2 Puromycin incorporation within isolated synaptosomes in basal and Aβ conditions. **(A**^**i**^**)** Synaptosomes were isolated from 24 DIV hippocampal neurons treated with vehicle or Aβ. Puromycilation assay was performed within isolated synaptosomes exposing them to 30 minutes of puromycin or puromycin co-incubated with anisomycin treatment. Scale bar 10 μm and 2 μm in insets. **(A**^**ii**^**)** Percentage (%) of puromycin puncta colocalization with Syn-Homer+ synapses, normalized to the total Syn-Homer+ synapses. Levels of no-puromycin are shown as negative control. Two-way ANOVA analysis with Holm-Sidak’s *post hoc* test for selected pairs of columns was performed, ***p*<0.01 and ns not significant. The graph represent the mean ± SEM of 5 independent biological experiments. **(B**^**i**^**)** Primary rat fibroblast cultures were characterized by staining with vimentin to label intermediate filaments (magenta), phalloidin for F-actin filaments (green) and DAPI (gray). Scale bar 50 μm. **(B**^**ii**^**)** Experimental protocol followed to performed puromycilation assay in isolated synaptosomes coming from neurons, neuron-fibroblast co-cultures or fibroblast conditioned medium (CM). The representative figure has been created with Biorender.

Supplementary Figure 3 Puro-Rpl26 PLA performed in synaptosomes from neuron-astrocyte co-cultures confirmed the inhibition of *Rpl26* synaptic local translation in Aβ conditions. Synaptosomes were isolated from 24 DIV vehicle- or Aβ-treated neuron cultures (N) or Aβ-treated neuron-astrocyte co-cultures (NA) in modified Boyden chambers were exposed 2-min with 2 μM puromycin. Anisomycin was used to block translation and no-puromycin treatment (- puromycin) as negative control. Puro-PLA of SNAP25 (SNAP25 PLA) representative micrographs are shown in **(A**^**i**^**)**. Scale bar 2 μm. Percentage (%) of the colocalization analysis obtained from SNAP25 PLA puncta with **(A**^**ii**^**)** synaptophysin-Homer+ synapses (obtained as a result of the double colocalization of Syn+ and Homer+ puncta), **(A**^**iii**^**)** Homer postsynapses (Homer+) and **(A**^**iv**^**)** Syn+ presynapses (Syn+). Each individual condition was normalized to the total amount of Syn-Homer+ synapses, Homer+ postsynapses or Syn+ presynapses, respectively. Puro-PLA of Rpl26 (Rpl26 PLA) representative micrographs are shown in **(B**^**i**^**)**. Scale bar 2 μm. Percentage (%) of the colocalization analysis obtained from Rpl26 PLA puncta with **(B**^**ii**^**)** synaptophysin-Homer+ synapses, **(B**^**iii**^**)** Homer+ postsynapses and **(B**^**iv**^**)** Syn+ presynapses. Each individual condition was normalized to the total amount of Syn-Homer+ synapses, Homer+ postsynapses or Syn+ presynapses, respectively. Data were analyzed in 5 independent cultures and analyzed by two-way ANOVA, ns not significant. All graphs represent mean ± SEM and the levels of no-puromycin negative controls are shown for descriptive purposes.

Supplementary Figure 4 Astrocyte-secreted factors do not have an impact on global synaptic SNAP25 and Rpl26 proteins. Total protein of SNAP25 and Rpl26 has been quantified within isolated synaptosomes coming from 24 DIV neuron or neuron-astrocyte co-cultures in basal condition. Percentage (%) of the colocalization of SNAP25 protein puncta with **(A**^**i**^**)** synaptophysin-PSD95+ synapses (obtained as a result of the double colocalization of Syn+ and PSD95+ puncta), **(A**^**ii**^**)** PSD95 postsynapses (PSD95+) and **(A**^**iii**^**)** Syn+ presynapses (Syn+). Each individual condition was normalized to the total amount of Syn-Homer+ synapses, PSD95+ postsynapses or Syn+ presynapses, respectively. Representative micrographs are shown in **(A**^**iv**^**)**. Scale bar 2 μm. Percentage (%) of the colocalization of Rpl26 protein puncta with **(B**^**i**^**)** synaptophysin-PSD95+ synapses, **(B**^**ii**^**)** PSD95+ postsynapses and **(A**^**iii**^**)** Syn+ presynapses. Each individual condition was normalized to the total amount of Syn-PSD95+ synapses, PSD95+ postsynapses or Syn+ presynapses, respectively. Representative micrographs are shown in **(B**^**iv**^**)**. Scale bar 2 μm. Data were analyzed in 5 independent cultures and analyzed by unpaired *t*-test, ns not significant.
